# Pre- and Postoperative Vomiting in Children Undergoing Video-Assisted Gastrostomy Tube Placement

**DOI:** 10.1155/2014/871325

**Published:** 2014-08-12

**Authors:** Torbjörn Backman, Helén Sjövie, Malin Mellberg, Anna Börjesson, Magnus Anderberg, Carl-Magnus Kullendorff, Einar Arnbjörnsson

**Affiliations:** ^1^Department of Paediatric Surgery, Skåne University Hospital, 221 85 Lund, Sweden; ^2^The Institution of Clinical Research, Lund University, 221 85 Lund, Sweden

## Abstract

*Background*. The aim of this study was to determine the incidence of pre- and postoperative vomiting in children undergoing a Video-Assisted Gastrostomy (VAG) operation. *Patients and Methods*. 180 children underwent a VAG operation and were subdivided into groups based on their underlying diagnosis. An anamnesis with respect to vomiting was taken from each of the children's parents before the operation. After the VAG operation, all patients were followed prospectively at one and six months after surgery. All complications including vomiting were documented according to a standardized protocol. *Results*. Vomiting occurred preoperatively in 51 children (28%). One month after surgery the incidence was 43 (24%) in the same group of children and six months after it was found in 40 (22%). There was a difference in vomiting frequency both pre- and postoperatively between the children in the groups with different diagnoses included in the study. No difference was noted in pre- and postoperative vomiting frequency within each specific diagnosis group. *Conclusion*. The preoperative vomiting symptoms persisted after the VAG operation. Neurologically impaired children had a higher incidence of vomiting than patients with other diagnoses, a well-known fact, probably due to their underlying diagnosis and not the VAG operation. This information is useful in preoperative counselling.

## 1. Introduction

Gastrostomy is advocated for children with long-term nutritional feeding problems exceeding 3 months, independent of the underlying diagnosis. The percutaneous endoscopic gastrostomy (PEG) technique has been the most widely used method to achieve enteral access since its introduction in 1980 [1]. Due to an association between the PEG procedure and serious complications [2, 3] such as gastroenteric fistulas, we have, at our Department of Paediatric Surgery, chosen to use and further develop the Video-Assisted Gastrostomy (VAG) technique since 1994 [4–6]. The advantages of the VAG technique over PEG are better visual control intra-abdominally, the secure positioning of the stoma to the gastric wall, and the gastroscopic control of the gastrostomy button placement in the ventricle cavity [3, 4, 7–9]. Many centres now use the laparoscopic technique because it is considered to be a safer method [10, 11].

Vomiting is a natural physiological process in children and is associated with gastroesophageal reflux (GER) and sometimes with gastroesophageal reflux disease (GERD) [12]. Whether gastrostomies lead to exacerbation of GERD has been debated, but guidelines and consensus nowadays dismiss that idea [13, 14], with the exception of the neurologically impaired children. Previously published materials concerning children operated on with VAG and undergoing oesophageal 24 h pH measurements pre- and postoperatively have not shown an increased acid reflux after the operation [5]. In a systematic review of GERD after the PEG procedure [15], the authors concluded that there was no current evidence that PEG insertion improved or aggravated GERD. Similar systematic reviews studying the relationship between VAG and GERD are not known to us. The aim of this study was to prospectively determine the incidence of vomiting pre- and postoperatively in children undergoing a VAG operation.

## 2. Material and Methods 

From June 2006 to December 2011, 180 children underwent a VAG operation as a single operative intervention. All are included in this study. The median (range) age was 1 year (1 month–14 years) and their body weight was 9 kg (3–41). During the period of the study, 33 children with cerebral palsy underwent a fundoplication. These were not included in this study.

The indication for a gastrostomy was nutritional problems in severely ill or neurologically impaired children when the need for nutritional support was considered necessary for more than three months. The operation was carried out only if the child's condition allowed safe operative intervention.

The work-up included an upper GI X-ray to rule out gastric outlet obstruction. All the patients and their parents were interviewed by a doctor and a specialized nurse and clinically evaluated for GERD before the VAG operation. The vomiting symptoms were recorded according to a standardized protocol and added to the patient's case notes. The protocol documented the children's problem including vomiting, tube dislodgement, granuloma formation, infection, and leakage. Endoscopy and 24 h pH measurements were carried out when an antireflux operation seemed indicated. No operations were performed prophylactically.

The VAG operation was performed as described in previously published articles [4, 11, 14]. Preoperative antibiotic prophylaxis was given. Through a lower umbilical skin incision, a minilaparotomy was done. A 3 mm VersaStep trocar was safely inserted in the abdomen and pneumoperitoneum was established with CO_2_ insufflation. Using a 3 mm 30-degree laparoscope, the ventricle was identified. In between the left costal margin and the umbilicus, a skin incision was made destined for the gastrostoma. A 5 mm trocar was inserted at this point into the abdomen under visual control with the laparoscope. Through this port, the ventricle was grasped with an instrument at its major side, with clear margins from the pylorus, and exteriorized when the grasper and trocar were pulled back. The ventricle was then sutured to the rectus muscle fascia and the gastrostomy tube was inserted into the cavity of the ventricle through a small incision in the ventricle wall. The placement of the gastrostomy tube was then controlled gastroscopically at the end of the surgical procedure.

All the children commenced oral feeding immediately postoperatively. Depending on the child's postoperative condition, a varying percentage of the total feeding was given through the gastrostomy. All the children were followed up prospectively during the first postoperative days in the hospital and at one and six months after surgery at our day care unit. Specialized nurses recorded all complications during the follow-up according to a standardized protocol. The endpoint of the study was at six months. Additional follow-ups were performed at any time on request from the parents. Vomiting was documented pre- and postoperatively according to the parents' reports with regard to the frequency and change in pattern or frequency of vomiting during the follow-up.

## 3. Statistical Analysis

Data were analysed by Fisher's exact probability test, two tailed, as well as nonparametric tests including the Mann-Whitney* U* test. A *P* value less than 0.05 was considered significant.

## 4. Ethical Consideration

The regional research ethics committee approved the study (registration number 2010/49). Permission to create a register of the patients was thereby obtained in accordance with the Swedish Privacy Protection Law.

## 5. Results

All 180 patients included in the study were operated on with the VAG method using the double U-stitch technique [6]. All the children tolerated the procedure. The development of granulation tissue, vomiting, infection, leakage, and tube dislodgement were the most common postoperative complications. Most of the patients had full resolution of their complications at six months after surgery (*P* < 0.05) except vomiting and tube dislodgement.

The incidences of vomiting pre- and postoperatively are summarized in Table 1. In the group of 51 children with preoperative vomiting symptoms, 43 (84%) had symptoms one month after surgery and 40 (78%) had remaining symptoms at the endpoint of the study, 6 months postoperatively. Of the children who had no preoperative vomiting symptoms (*n* = 129), 14 (11%) had a gradual increase in vomiting frequency during the follow-up.

The children's underlying diagnoses and the vomiting frequencies in each group of children with the various diagnoses pre- and postoperatively and at 6 months of follow-up are listed in Table 2. There were differences in vomiting frequencies between the patient groups based on the underlying diagnosis both pre- and postoperatively (*P* = 0.0152 and *P* = 0.0049). There was, however, no difference in vomiting frequency pre- and postoperatively within the same patient group.

## 6. Discussion

The results of this prospective study of 180 consecutively VAG-operated children show that vomiting is a common symptom both pre- and postoperatively. Vomiting was one of the exceptions that most of the patients had full resolution of their minor complications at six months after surgery. Throughout the study period, the recording of vomiting frequency has been based on the parents' observations and therefore the interpretation of the results may be queried.

There was no definition of volume required for it to be called vomiting and no registration during hospitalization or at the outpatient clinic during follow-up as to how many times/day the patients vomited. But since the same parents have observed vomiting frequency in the same patients, we consider the results to be reliable. It is, however, possible that a more objective definition and registration of vomiting might have led to different results regarding vomiting frequency.

It is difficult to quantify vomiting. The children's guardians can describe whether the child just throws up a small amount of food when he/she has eaten too much for that moment, which we do not classify as vomiting, or whether the vomiting really does not have any direct correlation to the intake of food and affect the child's wellbeing.

This study also shows that the problem of preoperative vomiting persists in most of the children after a VAG operation. In the group of children with preoperative vomiting, 78% still vomited at six months postoperatively. These findings might indicate that the operation itself does not considerably influence the vomiting symptom but that the reason why it persists is more likely to be found in the children's underlying diagnoses.

The question whether this study adds anything new to the literature is reasonable. It could be argued that while PEG and VAG are different in terms of procedure and complications, the physiology caused by them would be the same. However, there are some differences in the procedures. Performing the VAG operative intervention, the operating surgeon is always sure of where the gastrostomy is placed on the stomach, that is, on the anterior stomach wall. When performing the PEG operation on children, the gastrostomy sometimes happens to be placed on the posterior wall of the stomach. This is a consequence of the blind puncture through the abdominal wall and filling the stomach with air during the PEG procedure. Thus, there are some minor differences between the PEG and VAG procedures that theoretically may have physiological consequences since the stomach may be twisted clockwise during the PEG procedure.

In the group of patients that did not vomit preoperatively, there was a significant increase in vomiting frequency of 1.5% at one month and 11% at six months postoperatively. These findings cannot be traced to a certain diagnosis or age group. The reason or reasons behind this increase in vomiting frequency can only be speculated upon. One possible reason might be placement of the gastrostomy button close to the pylorus, causing a slight gastric outlet obstruction. This was not, however, noted or commented upon during the gastroscopy at the end of the VAG procedure. Changes with time due to the child's growth or the movements of the stomach cannot be ruled out as a possible explanation to the increase in vomiting with time in this subgroup of children. Another potential explanation for the increased vomiting frequency might be a general deterioration in the child's underlying disease. This group might then be equalled with the group with preoperative vomiting; that is, the cause is to be found in their underlying diagnosis and not in the VAG procedure.

There was a difference in vomiting frequency between the patient groups based on their underlying diagnosis both pre- and postoperatively. Vomiting occurred more frequently in the neurologically impaired children than in the other groups. The same finding has been described earlier in the literature but then as a part of GER symptom [12–15]. There was, however, no difference in vomiting frequency pre- and postoperatively within each specific group.

Even with the knowledge of the vomiting frequencies seen in this study, the VAG operation is still beneficial for many of the children and their families. Protracted forced meals orally or frequent replacement of nasogastric tubes without any secure weight gain causes a lot of frustration for the children and their parents. The vomiting frequency might not change with a gastrostomy, but the ease in nutritional support that comes with it often compensates for the vomiting. Preoperative information to the parents about our experience for their understanding of eventual future complications is a keystone for a successful postoperative result.

## 7. Conclusion

Vomiting, observed preoperatively, persisted six months postoperatively in 78% of the patients; its cause is most likely to be found in the children's underlying diagnosis and not in the VAG procedure. These conclusions can be useful information for parents and colleagues when counselling before a VAG operation answering the question about the fate of the child's problems with vomiting.

## Figures and Tables

**Table 1:** Vomiting pre- and postoperatively after operation using the VAG technique in 180 children.

Vomiting preoperatively	Vomiting postoperatively	1 month	6 months	*P* value∗
*N* = 180				
Yes 51	Yes	43	40	0,6119
	No	8	11	

No 129	Yes	2	14	0,0032
	No	127	115	

∗Statistical method: Fisher's exact probability test.

**Table 2:** The diagnosis of the included 180 patients and their vomiting frequency pre- and postoperatively at study endpoint at 6 months. The children had comorbidity in the form of epilepsy in 27 (17%), ventricular-peritoneal shunt in 9 patients (5%), and mitochondrial disease in 6 patients (4%).

Diagnosis	*N* (%)	Vomiting preoperatively *N* (%)	Vomiting postoperatively *N* (%)	*P* value∗
Cerebral pares	61 (34)	12 (24)	14 (35)	0,8254
Cardiac malformation	30 (17)	11 (22)	7 (18)	0,3985
Metabolic disease	37 (21)	10 (20)	5 (13)	0,2470
GI malformations	7 (4)	2 (4)	1 (3)	1
Malignancy	11 (6)	2 (4)	2 (5)	1
Respiratory insufficiency	15 (8)	7 (14)	6 (15)	1
Syndrome	19 (10)	7 (14)	5 (13)	0,7290
Sum	180 (100)	51 (100)	40 (100)	0,2253
*P* value∗∗		*P* = 0,0152	*P* = 0,0049	

Statistical methods:

∗Fisher's exact probability test, two tailed,

∗∗Mann-Whitney *U* test.

## References

[1] Gauderer WL, Ponsky JL, Izant RJ (1980). Gastrostomy without laparotomy: a percutaneous endoscopic technique. *Journal of Pediatric Surgery*.

[2] Khattak IU, Kimber C, Kiely EM, Spitz L (1998). Percutaneous endoscopic gastrostomy in paediatric practice: complications and outcome. *Journal of Pediatric Surgery*.

[3] Lantz M, Larsson HH, Arnbjörnsson E (2010). Literature review comparing laparoscopic and percutaneous endoscopic gastrostomies in a pediatric population. *International Journal of Pediatrics*.

[4] Andersson L, Mikaelsson C, Arnbjörnsson E, Larsson LT (1997). Laparoscopy aided gastrostomy in children. *Annales Chirurgiae et Gynaecologiae*.

[5] Plantin I, Arnbjörnsson E, Larsson L (2006). No increase in gastroesophageal reflux after laparoscopic gastrostomy in children. *Pediatric Surgery International*.

[6] Backman T, Sjövie H, Kullendorff CM, Arnbjörnsson E (2010). Continuous double U-stitch gastrostomy in children. *European Journal of Pediatric Surgery*.

[7] Arnbj÷rnsson E, Backman T, Mörse H, Berglund Y, Kullendorff CM, Lövkvist H (2006). Complications of video-assisted gastrostomy in children with malignancies or neurological diseases. *Acta Paediatrica*.

[8] Arnbjörnsson E, Larsson LT, Lindhagen T (1999). Complications of laparoscopy-aided gastrostomies in pediatric practice. *Journal of Pediatric Surgery*.

[9] Backman T, Arnbjörnsson E, Berglund Y, Larsson L (2006). Video-assisted gastrostomy in infants less than 1 year. *Pediatric Surgery International*.

[10] Akay B, Capizzani TR, Lee AM (2010). Gastrostomy tube placement in infants and children: is there a preferred technique?. *Journal of Pediatric Surgery*.

[11] Jones VS, La Hei ER, Shun A (2007). Laparoscopic gastrostomy: the preferred method of gastrostomy in children. *Pediatric Surgery International*.

[12] Hegar B, Vandenplas Y (2013). Gastroesophageal reflux: natural evolution, diagnostic approach and treatment. *The Turkish Journal of Pediatrics*.

[13] Vandenplas Y, Rudolph CD, Di Lorenzo C (2009). Pediatric gastroesophageal reflux clinical practice guidelines: joint recommendations of the North American Society for Pediatric Gastroenterology, Hepatology, and Nutrition (NASPGHAN) and the European Society for Pediatric Gastroenterology, Hepatology, and Nutrition (ESPGHAN). *Journal of Pediatric Gastroenterology and Nutrition*.

[14] Sherman PM, Hassall E, Fagundes-Neto U (2009). A Global, evidence-based consensus on the definition of gastroesophageal reflux disease in the pediatric population. *American Journal of Gastroenterology*.

[15] Noble LJ, Dalzell AM, El-Matary W (2012). The relationship between percutaneous endoscopic gastrostomy and gastro-oesophageal reflux disease in children: a systematic review. *Surgical Endoscopy and Other Interventional Techniques*.

